# Classes, Databases, and Prediction Methods of Pharmaceutically and Commercially Important Cystine-Stabilized Peptides

**DOI:** 10.3390/toxins10060251

**Published:** 2018-06-19

**Authors:** S M Ashiqul Islam, Christopher Michel Kearney, Erich Baker

**Affiliations:** 1 Institute of Biomedical Studies, Baylor University, Waco, TX 76798, USA; S_islam@baylor.edu; 2 Department of Biology, Baylor university, Waco, TX 76798, USA; Chris_Kearney@baylor.edu; 3 Department of Computer Science, Baylor university, Waco, TX 76798, USA

**Keywords:** cystine-stabilized peptides, databases, prediction tools, knottins, cyclotides, STPs, NTPs

## Abstract

Cystine-stabilized peptides represent a large family of peptides characterized by high structural stability and bactericidal, fungicidal, or insecticidal properties. Found throughout a wide range of taxa, this broad and functionally important family can be subclassified into distinct groups dependent upon their number and type of cystine bonding patters, tertiary structures, and/or their species of origin. Furthermore, the annotation of proteins related to the cystine-stabilized family are under-represented in the literature due to their difficulty of isolation and identification. As a result, there are several recent attempts to collate them into data resources and build analytic tools for their dynamic prediction. Ultimately, the identification and delivery of new members of this family will lead to their growing inclusion into the repertoire of commercial viable alternatives to antibiotics and environmentally safe insecticides. This review of the literature and current state of cystine-stabilized peptide biology is aimed to better describe peptide subfamilies, identify databases and analytics resources associated with specific cystine-stabilized peptides, and highlight their current commercial success.

## 1. Introduction

Cystine-stabilized peptides represent a large family of peptides characterized by high structural stability [1] and bactericidal [2], fungicidal [3], or insecticidal [4,5] properties. They are ubiquitous among a wide range of taxa and derive their functional characteristics from primary peptide sequence patterns that create distinctive tertiary structures and functional capacities [6]. Furthermore, the molecular structure of cystine-stabilized peptides lends to their popular commercial characteristics: stability [7], biosafety [8], and specificity [9]. If developed commercially, these naturally occurring toxins have the potential to serve as substitutes for antibiotics and insecticides that carry liabilities such as the development of pathogen/insect resistance and environmental toxicity [10].

Cystine-stabilized peptides have predominantly been studied in arthropods, but these peptides are also abundantly present in other taxa [5,11,12]. While traditional prediction methods relying on the isolation and identification of proteins from the environment are adequate, they are expensive and time-consuming. If cystine-stabilized peptides are to be exploited for broader use, more emphasis must be placed on their rapid prediction. However, the physical confirmation and lower penetrance of proteins belonging to this group immensely complicate their prediction from primary sequence [13]. The rapid adaptation of machine learning in this space is allowing us to compress the time between protein annotation and commercial development to a point where they will soon achieve their maximum impact. 

Here, we present a current view of the rapidly changing landscape of cystine-stabilized peptides. This includes a description of three major subfamilies (sequential tri-disulfide peptides [13], knottins [7], and cyclotides [14]), a discussion of source organisms for this class of peptides, and a list of resources for cystine-stabilized protein prediction, collections, and utility. In addition, we identify cystine-stabilized peptides that have achieved commercial viability and highlight other potential properties that will enhance future development. 

## 2. Cystine-Stabilized Peptide Families

**Figure 1:**
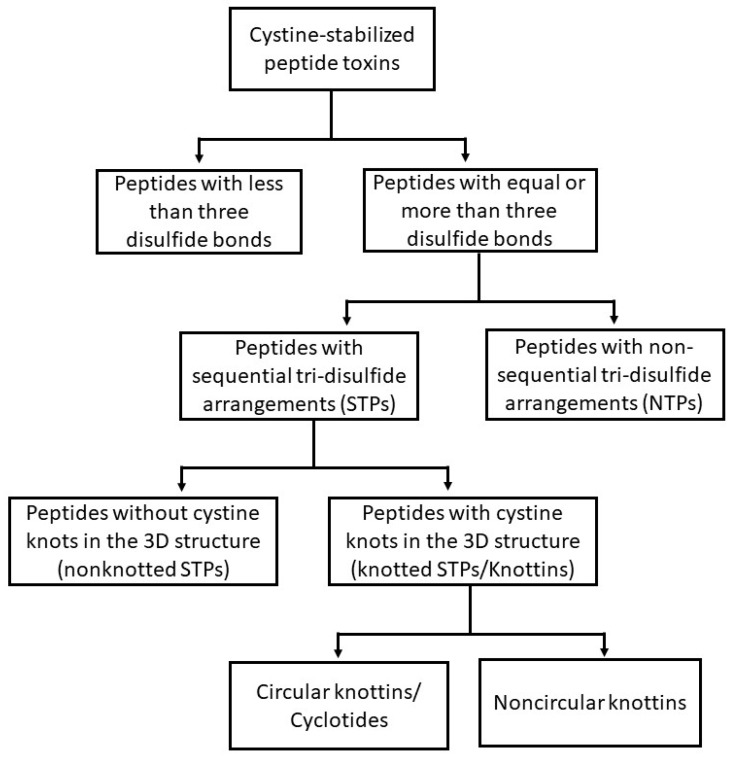
Classification structure of cystine-stabilized peptides based on intrachain connectivity.

**Figure 2:**
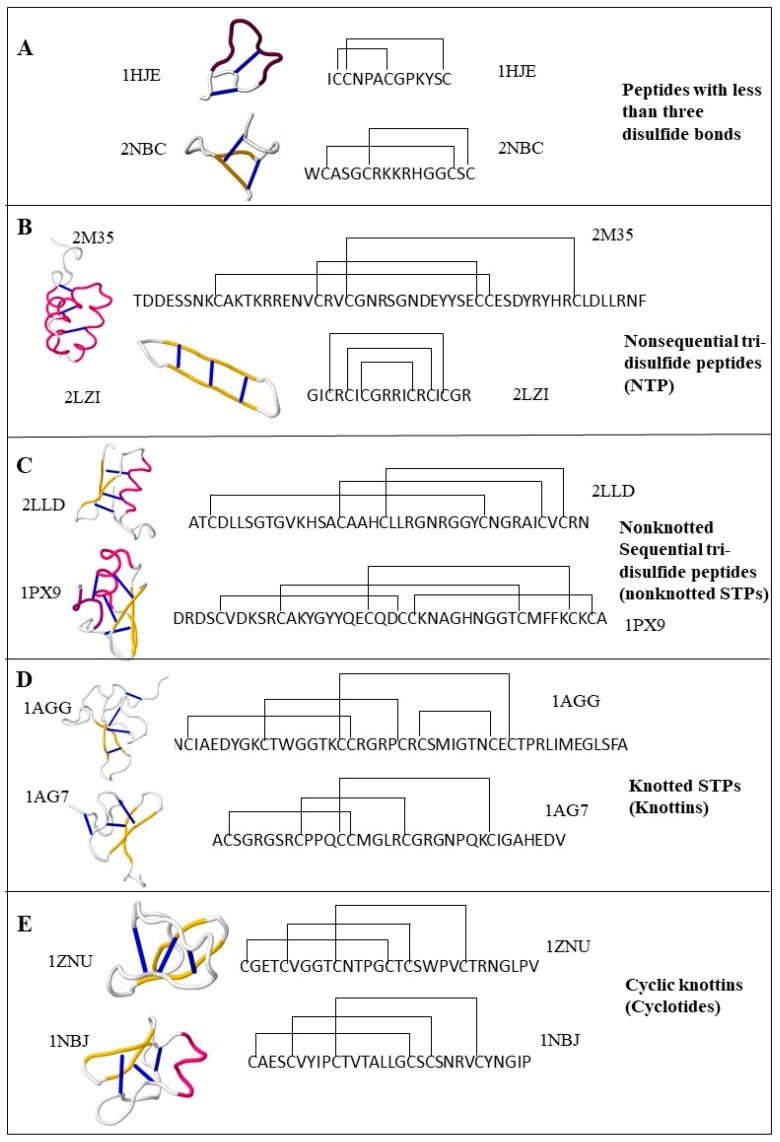
The structure of representatives of each intrachain connectivity-based groups with corresponding disulfide connectivity. Each block of the figure illustrates examples drawn from each group. The four-letter codes indicate the PDB (Protein Data Bank) IDs of the structures and sequences. Panel (**A**–**E**) illustrates the representatives of “peptides with fewer than three disulfide bonds,” nonsequential tri-disulfide peptides (NTPs), nonknotted sequential tri-disulfide peptides (STPs), Knottins, and Cyclotides, respectively.

This review describes cystine-stabilized peptide families based on disulfide bonding patterns or biological source and function. The classification schema outlined here is presented in Figure 1, with example peptides from each family provided in Figure 2.

### 2.1. Cystine-Stabilized Peptide Families Based on Disulfide Bonding Patterns

#### 2.1.1. Peptides with Three or More Disulfide Bonds

**Figure 3:**
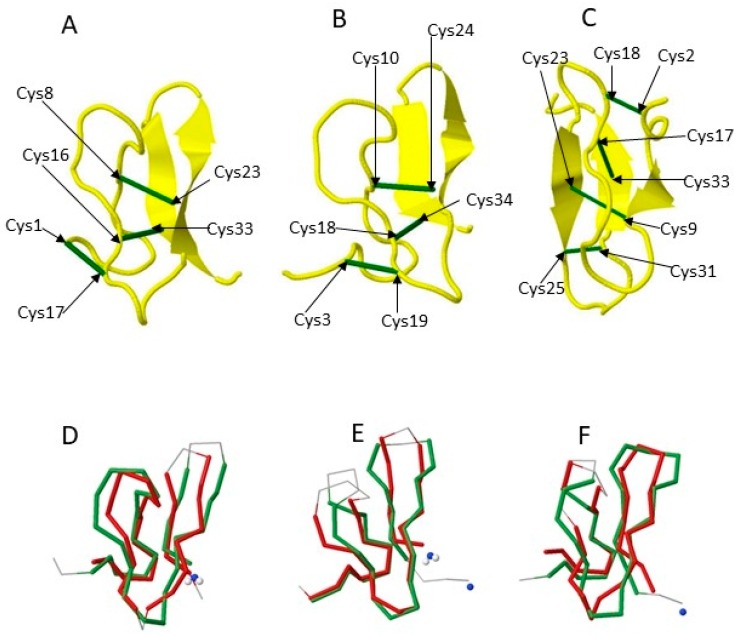
Similar structures may have differing sequence similarity and differing functions. Illustrated are three sequential tri-disulfide peptides (STPs) of similar structure (upper panel) and pairwise 3D alignments (lower panel). In the upper panel (**A**–**C**), the green lines represent the disulfide bonds. (**A**) The 3D structure of Ep-AMP1 (PDB Id 2MFS), an antimicrobial peptide isolated from cactus; (**B**) The 3D structure of Agelenin (PDB Id 2E2S), an insecticidal calcium channel inhibitor isolated from the venom of spider; (**C**) The 3D structure of Delta-palutoxin IT2 (PDB Id 1V91) which is an insecticidal voltage-gated sodium channel inhibitor isolated from the venom of spider; (**D**) The pairwise structure alignment between Ep-AMP1 and Agelenin with a 71.33 score, 29% sequence identity and 38% sequence similarity; (**E**) The pairwise structure alignment between Agelenin and Delta-palutoxin IT2 with a 62.10 score, 35% sequence identity and 47% sequence similarity; (**F**) The pairwise alignment between Ep-AMP1 and Delta-palutoxin IT2 with a 44.52 score, 37% percent sequence identity and 40% sequence similarity.

Three or more disulfide bonds allows the creation of a high level of variable peptide intrachain disulfide connectivity. As a group, these peptides represent a diverse set of distinctive bonding patterns denoted in the literature as sequential tri-disulfide peptides (STPs), nonsequential tri-disulfide peptides (NTPs), knottins, and cyclotides. The structure and function of this group of peptides represents a wide variety of differing structures and functional characteristics (Figure 2 and Figure 3).

#### 2.1.2. Sequential Tri-Disulfide Peptides (STPs) and Nonsequential Tri-Disulfide Peptides (NTPs)

STPs represent the group of cysteine-rich proteins that contain at least three disulfide bonds in a sequential pattern [13]. More explicitly, these peptides have a six cysteine motif in the primary sequence offering a disulfide bonding pattern between C1–C4, C2–C5, and C3–C6, where “C” represents the amino acid cysteine. The secondary structure of this group of peptides contains two or more beta sheets, beta hairpins, or cystine-stabilized alpha-beta sheets. Peptides with diverse functional characteristics, such as ion channel blockers [15], antimicrobial peptides [11,16,17], serine protease inhibitors [18], acetylcholine receptor inhibitors [19], and hemolytic peptides [20], contain this disulfide bonding pattern. The m-NGSG-based PredSTP algorithm [21] estimated that 69.20% of all deposited cystine-stabilized sequences are STPs. 

There are some peptides that have at least three disulfide bonds and show similar structural and functional characteristics without having an STP disulfide connectivity pattern [13]. This group of the proteins are defined as NTPs in the literature. NTPs are spread through a wide range of taxa and function as defensins, bacteriocins, and channel blockers. There is no dedicated database for STPs or NTPs; however, an SVM-based (support vector machine) machine learning model is available to predict STPs with high accuracy [13]. The second and third rows of Figure 2 illustrates examples of NTPs and STPs, respectively. 

#### 2.1.3. Knottins (Knotted STPs)

Knottins, or ICK (Inhibitor Cystine Knots), are the most well-known type of the cystine-stabilized peptides. These peptides have the canonical STP bonding pattern of C1–C4, C2–C5, C3–C6. However, while folding, the third disulfide bond penetrates through the first two disulfide bonds creating a cystine knot. This structure indicates that this group is a subset of the STP group. 

ICK peptides have been commercially developed and have different functional characteristics and medical/agricultural applications, such as neurotransmitters, analgesics, anthelmintics, anti-erectile dysfunction, antimalarials, antimicrobials, antitumor agents, protease inhibitors, toxins, and insecticides [7]. To offer a proper representation and documentation of the functional annotation and bibliographic data, a well-curated database, KNOTTIN, was developed as a dedicated repository for ICK peptides [22]. Protein data bank and Uniprot also included “knottin” as a structural motif in their database. In addition, tools to predict knottins from the primary and 3D structure of proteins are included in the Knoter 1D database and Knoter 3D database, respectively. The forth row of Figure 2 illustrates examples of knottins. 

#### 2.1.4. Cyclotides (Cyclic Knottins)

Cyclotides are a subset of knottins that have a head-to-tail cyclic backbone. The combination of cystine knot and cyclic backbone is also known as a cyclic cystine knot. Kalata B1, from the plant *Oldenlandia affinis* (*Rubiaceae*), was the first cyclotide described and was experimentally confirmed as containing a cystine knot with the macrocyclic structure in 1995. However, the peptide itself was first discovered in the early 1970s as an active ingredient of uterotonic, which is used traditionally as a boiled tea to accelerate childbirth [23]. The harsh means of preparation of the uterotonic remedy revealed the stability of Kalata B1, which was later found to be highly resistant to high temperatures and digestive enzymes [23]. Since then, cyclotides have been found to comprise a functionally diverse group of defense peptides, including insecticidal [24,25], nematicidal [26,27,28,29], and molluscicidal [29] peptides. 

Cyclotides can be formally divided into three groups: the Möbius, the bracelet, and trypsin inhibitor subfamilies. Kalata B1, Cycloviolacin O2, and the inhibitor MCoTI-II are specific to these groups, respectively. The bracelet and Möbius cyclotide folds are similar to each other, with the main difference occurring in Loop 5, where a *cis* tryptophan–proline bond results in a 180° twist of the peptide backbone Ω-angle of the Möbius fold [30]. On the other hand, trypsin inhibitors have entirely different peptide sequences from the other groups, with a more extended sequence in Loop 1. The coffee and violet plant families *Rubiaceae* and *Violaceae* are the source of the Möbius and Bracelet type of cyclotides, respectively [31,32,33], while the third type, trypsin inhibitors, were isolated from the melon family *Cucurbitaceae* [34,35]. A few cyclotides are also found in the bean, potato, and grass families *Fabaceae*, *Solanaceae*, and *Poaceae*, respectively [36,37,38,39]. Cybase was developed to facilitate the search and display of cyclic proteins for functional and structural analysis [40]. A significant fraction of the documented proteins in Cybase are cyclotides or other cystine-stabilized cyclic peptides such as theta defensin RTD-1 [41] and cyclic bacteriocin [42]. 

As of January 2018, A total of 314 cyclotides were documented in Cybase. However, it is conservatively accepted that a significant fraction of the cyclotides remain unannotated. CyPerl and CyExcel are two BLAST independent tools invented for the prediction of cyclotide analogs from plant genome and other protein databases [43]. A total of 202 novel cyclotide analogs were harvested from seven different plant families using CyPerl and CyExcel tools. Cypred is a machine leaning-based sequence alignment-independent predictor for cyclic peptides. Utilizing a test set, Cypred offers a 98.7% percent accuracy which is better than the accuracy calculated using BLAST and pairwise sequence alignment on the same test set. CycloMod is another tool to predict the 3D structure of a putative cyclic protein as a PDB format [40]. The fifth row of Figure 2 illustrates examples of cyclotides.

#### 2.1.5. Peptides with Fewer than Three Disulfide Bonds

While few cystine-stabilized toxins contain fewer than three disulfide bonds, a wide range of organisms express them as peptides. Many antimicrobial peptides [44,45] and channel blockers [46] fall into this group; see the first row of Figure 2. No database has been reported for this group of peptides; however, CSPred is a machine learning based software that can be used to predict the functional properties of this group of peptides along with other cystine-stabilized peptides. CSPred is discussed more in the “Function-Based in silico Classification of Cystine-Stabilized Peptides” section of this article.

### 2.2. Source-Based Subfamilies of Cystine-Stabilized Peptides

#### 2.2.1. Spider and Scorpion Toxins

A large fraction of cystine-stabilized bioactive peptides consists of spider venom peptides. Spiders use their toxins to kill or paralyze their prey via molecular interference with the neurotransmission process. Besides general neurotoxicity, spider toxins also display antiparasitic, hemolytic, analgesic, cytolytic, antimicrobial, antiarrhythmic, and enzyme inhibitory activity [47]. The primary mode of actions of these toxins are interfering or binding with transporters, receptors, and carbohydrate of lectins, perturbing membrane and inhibiting different ion and other channels [15,48]. Arachnoserver [49] is a spider toxin database which contains a repository of 1426 spider toxins (January 2018). These are categorized based on taxonomy, molecular targets, post-translational modifications, and phyletic specificity. It is assumed that only a small fraction of the spider toxins has been discovered, and a significant portion of the listed toxins are unexplored experimentally. While there is no computational tool to detect spider toxins from the primary sequence, SpiderP is an available tool to predict the subcellular location of spider toxins using an SVM [50]. 

Another significant source of cystine-stabilized peptides are scorpion toxins. These can be divided into two categories based on chain size: long-chain and short-chain toxins. Most of the scorpion toxins interact with voltage-gated sodium and potassium channels. While the database dedicated to scorpion toxins, SCORPION2 [51], is no longer supported, the same research group has developed a tool to predict functional properties of scorpion toxins from the primary sequences [52].

#### 2.2.2. Conotoxins

Cone snails are a large reservoir of cystine-stabilized peptides, producing a wide array of toxin proteins in their venom gland [53]. These are designated “conotoxins,” and, as a group, they are primarily bioactive neurotoxins which are mainly divided into three subgroups: (1) voltage-gated ion channel blockers, (2) ligand-gated ion channel blockers, (3) and other receptor blockers [54]. Members of the third subgroup interact with neurotensin receptors, nicotinic acetylcholine, or G protein-coupled receptors (GPCRs) primarily. To facilitate the annotation procedure, signatures of different conotoxin families have been incorporated into PROSITE [55]. ConoServer [56] is a database constructed with the sequence, structure, and functional characteristics of conotoxins. As of January 2018, the number of entries in ConoServer consisted of 2838 nucleotide sequences, 6255 protein sequences, and 176 protein structures. The Conus Biodiversity website (http://biology.burke.washington.edu/conus/) is a phylogeny database which keeps a record of pictures and videos of different *Conus* species. Although a huge number of conotoxins are recorded in the database, it is commonly agreed that a significant fraction of conotoxins are still unexplored. 

To expedite discovery, a number of machine learning based algorithms have been reported which predict putative conotoxins from unknown primary protein sequences. In 2006, Mondal et al. proposed the first-ever conotoxin prediction model where features generated from PseAAC were used to train an SVM classifier with an 88.10% accuracy [57]. Later in 2007, an IDQD model that subclassifies conotoxins into superfamilies and families was developed with an accuracy of 87.7% and 72%, respectively [58]. Subsequently, the prediction and identification of conotoxins, which function as ion-channel inhibitors, have become mainstream. Likewise, a number of machine-learning–based algorithm using diverse feature extraction techniques from the primary protein sequences have been proposed within this scope [59,60,61,62]. Presence of these prediction models accelerates the identification of undiscovered conotoxins and the rapid extension of the conotoxin sequence coverage.

#### 2.2.3. Plant Cysteine-Rich Peptides

Plants serve as cystine-stabilized peptide factories, producing an enormous number of variations. These peptides are used primarily for defensive purposes [63] and include plant defensins [64], hevein-like peptides [65], crambins [66], lipid transfer proteins [67], knottin-like proteins [68], and snakins [69]. In addition, several plant pathogenesis-related proteins are cysteine-rich peptides. Pathogenesis-related proteins were first discovered in the early 1970s from tobacco leaves and operationalize around diverse mechanisms, such as antiviral, antifungal, antibacterial, chitinase, anti-oxidative activity, and proteinase inhibitory activities [70,71,72]. For example, cyclotide-type proteins in plants inhibit proteases, lipid transfer proteins bind to lipids and inhibit microbial infection into the cell membrane, and hevein-like peptides bind to chitins to defend the source plant from fungal infections. 

Based on common features extracted from thousands of plants genes, it is expected that the number of cystine-stabilized peptides in plants is underappreciated [73]. PhytAMP is a database of plant AMPs where a portion of the recorded proteins are plant cysteine-rich proteins [74]. The PhytAMP database contains information on the family, source organism, activity and target organisms for each of the total 273 entries. So far, there is no dedicated computational tool to discover plant cysteine-rich proteins from the primary sequence. However, an in silico method is available to predict hevein-like peptide precursors from the plant genome [65]. 

#### 2.2.4. Other Sources

In addition to previously discussed sources, cystine-stabilized are also available in bacteria, fungi, sea anemones, jellyfishes, echinoderms, snakes, lizards, fishes, and platypoids and, based on mounting evidence, in fleas, mosquitoes, kissing bugs, leeches, ticks, and vampire bats [75]. Bacteriocins are another group of peptides which are produced by bacteria to kill other bacteria. A substantial fraction of these are cystine-stabilized peptides, including laterosporulin [76] and thuricin CD [77], for example. Bacteriocins are becoming popular as antimicrobial peptides as the prevalence of antibiotic-resistant bacteria continues to rise. One advantage of using bacteriocins over other AMPs is that bacteriocins are specific to their target species and produce a minimal effect on commensal bacteria [78]. Bactbase is a database that is dedicated to bacteriocins; it also offers different bioinformatic tools to analyze bacteriocin peptide sequences [79]. 

Defensins are another group of cystine-stabilized peptides and are found in vertebrates [80], invertebrates [81] and plants [11]. These are mainly antimicrobial peptides active against fungi, bacteria, protists, and viruses. Unlike bacteriocins, defensins demonstrate activity against a broader range of bacteria, which often include commensals [80]. However, some fungal defensins have been engineered into targeted proteins recombinantly by attaching species-specific targeting domains. For example, plectasin [82] is a defensin from a pezizalean fungus which was modified to selectively kill methicillin-resistant *Staphylococcus aureus* (MRSA) when peptide ArgD from a quorum sensing system was attached to the N-terminus [9]. Islam et al. (unpublished) have achieved specfic targeting of plectasin [82] and eurocin [83] against *Staphylococcus* spp. by attaching a targeting moiety from a phage pathogen which specifically infects *Staphylococcus*. “Defensin Knowledbase” [84] is another database of interest for this class of peptides. It contains a record of the family, structure, target organism and the strength of activity for 566 defensins (January 2018). Importantly, it also contains the clinical records of 255 defensins. 

Cystine-stabilized peptides represent a wide array of functional importance, structural components, and organisms. While more work needs to be done to better understand their modes of action, their potential utility in pharmaceuticals and as alternatives to current antimicrobials is vast. As more members are annotated, the breadth of their application will continue to increase.

## 3. Function-Based In Silico Classification of Cystine-Stabilized Peptides

For commercial applications, the ability to classify peptide sequences by the peptide’s function is extremely useful. As a result, numerous databases dedicated to cystine-stabilized peptides have been created (Table 1) along with analytic tools for their classification and discovery (Table 2). For example, a large dataset of knottins might need to be screened to generate a short list of sequences corresponding to structurally stable sodium channel blockers. These could then be cloned in *E. coli*, expressed, and confirmed for stability and activity in the lab. In contrast, a traditional high-throughput method would chemically separate peptides from a single source, such as a venom, and then screen for a particular function, with success for only a small fraction of the many peptides screened. Thus, algorithms for function-based classification have practical value beyond a simple rational ordering of peptides. 

It is intriguing that peptides of highly similar structure can have strikingly different functions. Figure 3 illustrates the structural similarity between three knottins, one from a cactus and two from spiders. Though they share nearly identical structures in terms of the order and three-dimensional arrangement of loops and beta strands, these structures effect divergent strategies to achieve antimicrobial properties. The cactus peptide acts as calcium channel inhibitor, while the spider protein is a sodium channel inhibitor. By comparing patterns of sequence divergence of peptides of similar structure but divergent function, the location of common functional motifs may be discovered. There have been a limited number of algorithms developed for functional classification of peptides. Among these is the CSPred algorithm [85]. CSPred allows the classification of peptide primary sequences on the basis of predicted functionality, specifically, ion channel blockers, antimicrobial peptides, acetylcholine receptor inhibitors, serine protease inhibitors, and hemolytic proteins. The average reported accuracy of CSPred is around 92% using a five-fold cross-validation. 

## 4. Pharmaceutical and Commercial Applications

Several cystine-stabilized peptides have already been licensed for clinical or agricultural use and are listed in Table 3. This small fraction demonstrates the potential for new applications hidden among the thousands of unannotated cystine-stabilized peptide sequences in genomes across many taxa. A voltage-gated calcium channel blocker cystine-stabilized peptide (Hv1a) from spider venom [87] is now the primary product of Vestaron, Inc. (Kalamazoo, MI, USA), with commercial production in *E. coli* for broad-scale application on crops plants as an eco-friendly insecticide that degrades in the field within two weeks after spray application. This same spider peptide has been fused to a targeting moiety by another group to specifically target aphids as a transgene in plants [88]. In other studies, antimicrobial cystine-stabilized peptides have been targeted for specific toxicity against individual pathogenic bacterial species, with nontarget toxicity greatly reduced (Islam et al., unpublished data). This has implications for antibiotic treatment without the disruption of the native microbiome. 

Clinically, cystine-stabilized peptides have realized a diverse array of commercial applications. For example, alpha-bungarotoxin has a long history of use in isolating and identifying specific acetylcholine receptors and in the diagnosis of myasthenia gravis [89]. Aprotinin has been shown clinically effective against flu infection by inhibiting protease cleavage of HA0 to HA1 and HA2 [90] but is now mainly used to reduce bleeding. The calcium channel blocker from conch, ziconotide (Prialt), has been used clinically as a pain reliever [91]. The chloride channel blocker from scorpion, chlorotoxin, reached Phase III trials as a treatment for glioblastoma cancer [92,93]. Finally, Linaclotide, a cystine-stabilized peptide, is licensed for clinical use orally against irritable bowel syndrome [94]. 

## 5. Discussion

Cystine-stabilized peptides offer a vast opportunity for commercial development, for either clinical or industrial use. For example, the characteristics of structural stability, low cost and high biosafety have been fully actualized in the SPEAR™ series of insecticidal sprays currently marketed by Vestaron, Inc. This peptide formulation is stable enough to kill crop pest insects and yet labile enough to not persist in the environment like a traditional pesticide. This ideal has also been realized with Linaclotide, which is stable enough to survive the passage through the GI tract and be delivered as an oral drug. 

In other cases, the core strengths of cystine-stabilized peptides—stability, low cost, and biosafety—are insufficient and require new approaches to achieve commercial potential. For example, it was demonstrated that the native sequences of tested knottins were susceptible to digestion with gut proteases [95,96]. A variant form with mutated protease cleavage sites could provide a protease resistant knottin in this case. Alternatively, cyclotides, a knottin-subtype which have increased protease resistance due to their lack of N- and C-termini, may serve as practical substitutes [97]. In large scale applications, the cost of chemical synthesis of cystine-stabilized peptides may be prohibitive. This was solved by Vestaron, Inc., by optimizing *E. coli*-based production for field-scale production. An even less expensive approach is to incorporate cystine-based peptide sequences into the host as a transgene. 

Biosafety for toxins and antimicrobial peptides is mainly determined by specificity toward the intended target. Cystine-stabilized antimicrobial peptides are typically broadly toxic due to their generalized toxin targets, such as negatively charged bacterial membranes. This generalized targeting is beneficial in deferring the development of resistance by the bacteria, but it also confers toxicity against the entire microbiome, destroying commensal as well as pathogenic bacteria. Similarly, insecticidal cystine-stabilized peptides are often broadly insecticidal, killing pests as well as beneficial insects. A straightforward means of introducing specificity is required and may be achieved by genetically fusing targeting peptides to the cystine-stabilized peptide. This has been successfully demonstrated with peptides targeted to bacteria [98], fungi [99], and insects [100]. 

Data mining of cystine-stabilized sequences can bring a new approach to the successful development of this class of peptides for commercialization. By leveraging millions of years of evolutionary development for the success of environment-specific peptides and functions, for example, machine learning algorithms may identify antimicrobial peptides that are resistant to the specific proteases and chemical environments of the gut. Similarly, if a set of orally toxic insecticidal peptides are desired, it might make more sense to search the proteomes of plants rather than in spiders: those of the former being delivered orally in nature while those of the latter being delivered by injection (though it should be noted that orally toxic spider toxins are known). In this fashion, novel applications of cystine-stabilized peptides could be realized by thinking biologically and then using algorithms to powerfully reduce the candidate pool to a reasonable number before cloning and functional assays.

## Figures and Tables

**Table 1:** Databases of cystine-stabilized peptides.

Database Name	Target Protein	URL	Ref
Knottin	Knottins	http://www.dsimb.inserm.fr/KNOTTIN/	[22]
Cybase	Cyclotides and other disulfide rich cyclic proteins	http://www.cybase.org.au/	[40]
Arachnoserver	Spider toxins	http://www.arachnoserver.org	[49]
SCORPION2 *	Scorpion toxins	http://sdmc.i2r.a-star.edu.sg/scorpion/	[51]
ConoServer	Conotoxins	http://www.conoserver.org	[56]
PhytAMP	Cystine-rich antimicrobial peptides from plants	http://phytamp.hammamilab.org	[74]
Bactibase	Bacteriocins	http://bactibase.hammamilab.org	[79]
Defensin Knowledgebase	Defensins	http://defensins.bii.a-star.edu.sg	[84]

* The server is currently inactive.

**Table 2:** Cystine-stabilized peptide discovery and analysis tools.

Name of Software	Function	Web Link	Ref.
PredSTP	Predicts sequential tri-disulfide proteins (STP) from primary peptide sequence	http://crick.ecs.baylor.edu/	[13]
CSPred	Classifies cystine-stabilized peptides based on functional characteristics of the primary peptide sequence	http://watson.ecs.baylor.edu/cspred	[85]
Knoter 1D	Predicts knottins from primary peptide sequence	http://www.dsimb.inserm.fr/KNOTTIN/knoter1d.php	[22]
Knoter 3D	Knottin prediction from 3D protein structure	http://www.dsimb.inserm.fr/KNOTTIN/knoter3d.php	[22]
Cypred	Cyclic structure prediction from primary peptide sequence	http://biomine.cs.vcu.edu/servers/CyPred	[86]
Cyclomode	Cyclotide 3D structure prediction from primary peptide sequence	http://www.cybase.org.au/?page=cyclomod	[40]
SpiderP	Predicts the subcellular localization of spider toxins from the primary peptide sequence	http://www.arachnoserver.org/spiderP.html	[50]
iCTX-Type	Predicts subclasses of ion-channel binding conotoxins from the primary peptide sequence	http://lin-group.cn/server/iCTX-Type	[62]

**Table 3:** Commercialization of cystine-stabilized peptides.

Peptide	Structure	Company	Stage	Use
HXTX-Hv1a spider toxin	STP (knottin)	Vestaron	Commercial crop spray (SPEAR™ product line)	Control of thrips, whiteflies, caterpillars, beetles
Plectasin NZ2114	STP (defensin)	Novozyme (licensed to Sanofi-Aventis)	Phase I	Severe gram-positive bacterial infections
Brilacidin	Nonpeptide STP mimetic	Cellceutix	Phase II	Ulcerative proctitis
Linclotide	STP	Ironwood Pharmaceuticals	Phase III	Irritable bowel syndrome; chronic constipation
Ziconotide (Prialt)	STP (calcium channel blocker)	Azur Pharma	FDA approved; commercial	Analgesic
Alpha-bungaro-toxin	NTP (acetyl-choline receptor inhibitor)		Commercial	Diagnostics
Aprotinin	NTP (trypsin inhibitor)	Nordic Group Pharmaceuticals	Commercial	Reduction of bleeding
Chlorotoxin	STP (chloride channel inhibitor)	Transmolecular	Phase III	Anti-glioma and imaging
